# Dual Mode Action of Mangiferin in Mouse Liver under High Fat Diet

**DOI:** 10.1371/journal.pone.0090137

**Published:** 2014-03-05

**Authors:** Jihyeon Lim, Zhongbo Liu, Pasha Apontes, Daorong Feng, Jeffrey E. Pessin, Anthony A. Sauve, Ruth H. Angeletti, Yuling Chi

**Affiliations:** 1 The Laboratory for Macromolecular Analysis & Proteomics, Albert Einstein College of Medicine of Yeshiva University, Bronx, New York, United States of America; 2 Department of Pathology, Albert Einstein College of Medicine of Yeshiva University, Bronx, New York, United States of America; 3 Department of Medicine, Albert Einstein College of Medicine of Yeshiva University, Bronx, New York, United States of America; 4 Department of Molecular Pharmacology, Albert Einstein College of Medicine of Yeshiva University, Bronx, New York, United States of America; 5 Department of Pharmacology, Weill Cornell Medical College, New York, New York, United States of America; Hosptial Infantil Universitario Niño Jesús, CIBEROBN, Spain

## Abstract

Chronic over-nutrition is a major contributor to the spread of obesity and its related metabolic disorders. Development of therapeutics has been slow compared to the speedy increase in occurrence of these metabolic disorders. We have identified a natural compound, mangiferin (MGF) (a predominant component of the plants of *Anemarrhena asphodeloides and Mangifera indica*), that can protect against high fat diet (HFD) induced obesity, hyperglycemia, insulin resistance and hyperlipidemia in mice. However, the molecular mechanisms whereby MGF exerts these beneficial effects are unknown. To understand MGF mechanisms of action, we performed unbiased quantitative proteomic analysis of protein profiles in liver of mice fed with HFD utilizing ^15^N metabolically labeled liver proteins as internal standards. We found that out of 865 quantified proteins 87 of them were significantly differentially regulated by MGF. Among those 87 proteins, 50% of them are involved in two major processes, energy metabolism and biosynthesis of metabolites. Further classification indicated that MGF increased proteins important for mitochondrial biogenesis and oxidative activity including oxoglutarate dehydrogenase E1 (Dhtkd1) and cytochrome c oxidase subunit 6B1 (Cox6b1). Conversely, MGF reduced proteins critical for lipogenesis such as fatty acid stearoyl-CoA desaturase 1 (Scd1) and acetyl-CoA carboxylase 1 (Acac1). These mass spectrometry data were confirmed and validated by western blot assays. Together, data indicate that MGF upregulates proteins pivotal for mitochondrial bioenergetics and downregulates proteins controlling *de novo* lipogenesis. This novel mode of dual pharmacodynamic actions enables MGF to enhance energy expenditure and inhibit lipogenesis, and thereby correct HFD induced liver steatosis and prevent adiposity. This provides a molecular basis supporting development of MGF or its metabolites into therapeutics to treat metabolic disorders.

## Introduction

Metabolic disorders, including diabetes and liver steatosis, are currently epidemic, driven by increased prevalence of obesity as a result of sedentary lifestyles and high-calorie diets [1], [2]. Chronic over-nutrition has many adverse consequences, including mitochondrial dysfunction, hyperglycemia and hyperlipidemia. The molecular mechanisms of metabolic dysregulation leading to obesity and its related pathological conditions remain poorly understood, thus no effective therapy is currently available.

We and others have identified a natural compound, mangiferin (MGF), that could increase insulin sensitivity and mitigate hyperglycemia in diabetic animal models [3]-[5]. MGF is a major component of *Anemarrhena asphodeloides* and *Mangifera indica*, which produces mango [6]–[8]. In rats and hamsters, MGF was shown to be able to reverse HFD-mediated increases in cholesterol, triglyceride (TG), and low density lipoprotein (LDL) in plasma and liver [9], [10]. In humans, treatment with extract from African mango for over a period of 10 weeks resulted in significant weight loss of up to 12 kilograms in overweight and obese subjects, accompanied with improvements in total cholesterol, LDL and fasting blood glucose [59]. MGF or the extract containing MGF appears to exert several beneficial metabolic effects in animals and humans. However, the molecular mechanisms of MGF actions are poorly understood.

The liver plays a critical role in the integrative maintenance of whole body glucose and lipid metabolism through the dynamic control of lipogenesis, lipolysis, gluconeogenesis, and glycolysis. It is estimated that at any given time point more than 10,000 biochemical reactions occur in liver [11]. Those reactions include basic carbohydrate, fat and protein metabolism, storage of vitamins and minerals, and many regulatory processes that control blood lipid, sugar and hormone levels. More importantly, obesity related metabolic disorders manifest in liver, causing liver steatosis, nonalcoholic fatty liver disease, eventually leading to inflammatory steatohepatitis [2]. Studies have demonstrated that HFD has significant impacts on the global proteome in liver [11], [12]. We therefore speculated that MGF might modulate protein profiles in liver in a therapeutic positive manner to mitigate liver steatosis induced by HFD.

To address this issue, we took advantage of a proteomics approach that provides unbiased and comprehensive profiles of proteins. We have generated ^15^N metabolically labeled mice and performed quantitative analysis of proteins in liver of mice fed with HFD, with and without MGF added to the diet, utilizing the stable isotope labeling of mammalian (SILAM) livers as internal standards for cross comparison. Herein we report the impacts of MGF on the expression of proteins in liver and their implication for liver metabolic processes.

## Materials and Methods

### 2.1 Animal experiments

Wild type C57BL6/J mice at age of 5–6 weeks were purchased from the Jackson Laboratory. All experimental procedures done with mice were approved by the Institutional Care and Use Committee at Albert Einstein College of Medicine in accordance with the “Guide for the Care and Use of Laboratory Animals” published by the National Institute of Health. Chow diet (CD) (providing calories 28.5%, 13.5% and 58% from protein, fat and carbohydrate) was purchased from Purina LabDiet (Framingham, MA). HFD (providing calories 20%, 60% and 20% from protein, fat and carbohydrate) was purchased from Research Diets (New Brunswick, NJ). Higher than 95% pure MGF was purchased from CTMedChem (Bronx, NY). CD or HFD was grinded into fine powder and mixed with pure MGF at dose of 0.5% (g MGF/g food). The mixtures were re-pelleted and administered to individually housed mice. Mice were fed with food and water *ad libitum*. MGF was given for 18 weeks.

### 2.2 Analysis of lipids in plasma

TG, total cholesterol, LDL, high density lipids, alkaline phosphatase (ALP), alanine transaminase (ALT) and aspartate transaminase (AST) were analyzed by the Einstein Translational Einstein-Montefiore Institute for Clinical and Translational Research.

### 2.3 Oil Red O staining

Liver tissues were fixed in 10% neutral buffered formalin (Fisher Scientific) for at least 48 hours, followed by rapid freezing in Tissue Tek OCT (Sakura Finetek) for cryosectioning. Cryosections of 5 µm were stained by routine Oil Red O methods (Poly Scientific R&D Corp.) and lightly counterstained in Mayer's hematoxylin solution.

### 2.4 Generation of ^15^N metabolically labeled mice

^15^N metabolically labeled mice were prepared as previously described [13]. Briefly, C57BL6/J mice were fed with ^15^N-labeled specially fortified diet (Research Diets, Inc., New Brunswick, NJ) using ^15^N-labeled spirulina (Cambridge Isotope Laboratories, Tewksbury, MA) starting immediately after weaning (21 days of age) for a total period of 12 weeks. Metabolic labeling efficiency was monitored non-invasively by the analysis of urine sediment while the labeling process was ongoing. Greater than 95% atomic enrichment of liver proteins was achieved.

### 2.5 Preparation of samples for LC- MS/MS analysis

**Figure 1:**
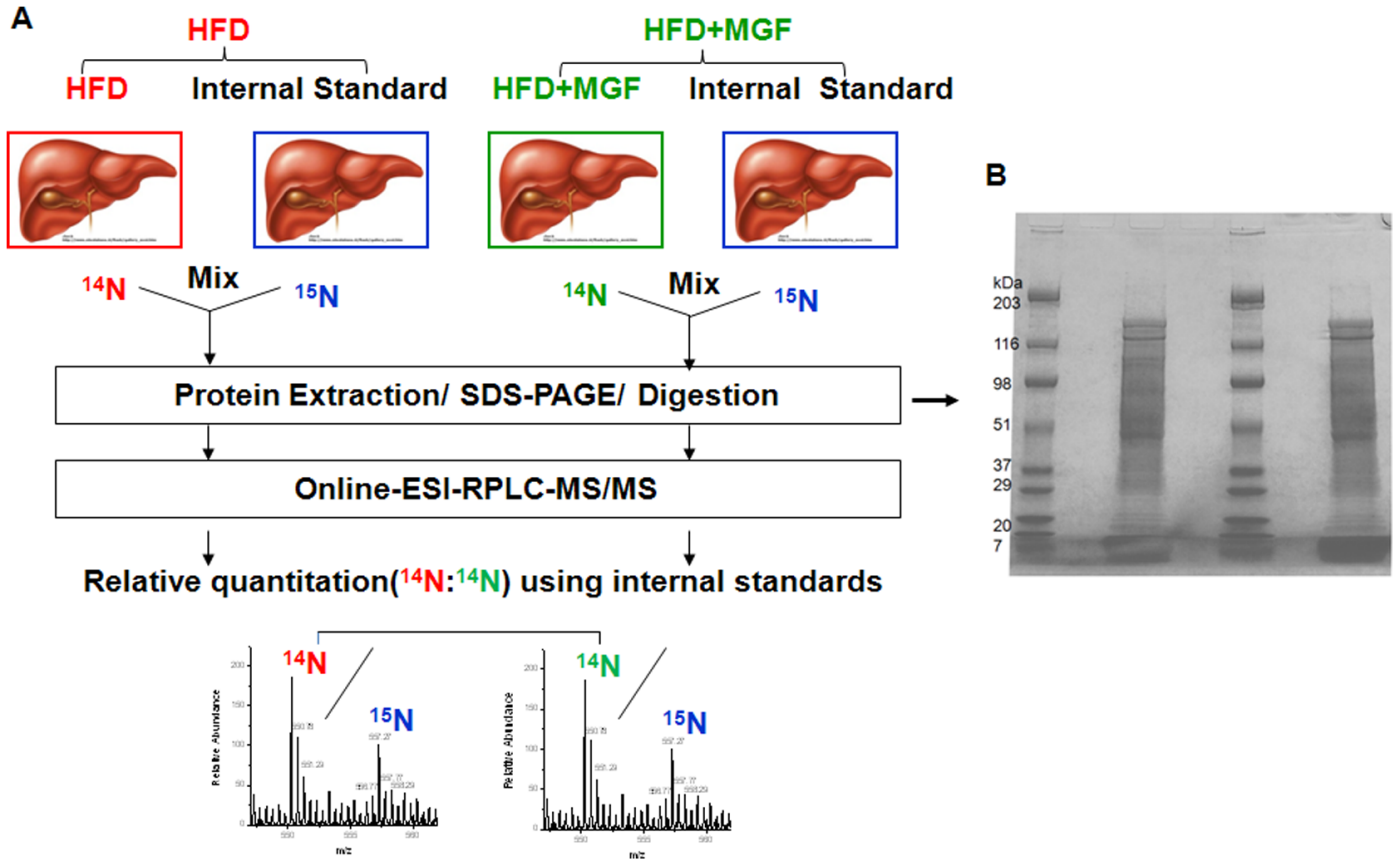
Workflow for analysis of differential expression of proteins upon MGF treatment. (A) Extracted proteins were separated on SDS-PAGE. Fractionated proteins were in-gel digested to produce tryptic peptides to be analyzed on the LC-MS/MS. Relative abundance of two groups (HFD and HFD + MGF) are extracted using heavy (^15^N) labeled peptides as internal standards. (B) One dimensional SDS-PAGE (Tis-HCl 4–15%) of samples. (MM: molecular marker, 50 mg of protein extracts from 1: HFD plus ^15^N mouse livers and 2: HFD+MGF plus ^15^N mouse livers).

Liver tissues from 4 HFD-fed mice were pooled and ground together. Matching livers from 4 HFD-MGF treated mice were prepared in the same manner. Each group of pooled livers was mixed with equal weight of liver tissue from ^15^N metabolically labeled mouse (Figure 1A). Tissues were homogenized and lysed in RIPA buffer with protease inhibitor cocktail for protein extraction. Fifty µg of extracted proteins were separated with SDS-PAGE (Figure 1B). Complete lanes were excised using disposable grid cutters (The Gel company, San Francisco, CA) to produce 40 bands per slice, band slices were placed in 96-well plates and in-gel digested using an Ettan Digester (GEHealthcare, Piscataway, NJ). Digested peptides from 2-3 bands were pooled before analysis. The samples from the duplicate experiments with 4 additional mice in each group (HFD ± MGF) were obtained using the same method.

### 2.6 LC- MS/MS analysis

The Agilent 1100 Series Nano HPLC interfaced to a QStar XL mass spectrometer (AB Sciex, Ontario, Canada) was used for analysis. Samples were loaded onto a ZORBAX 300SB-C18 trap column (5 µm, 300 Å, 0.3 mm) at a flow rate of 8 µL/min with 2% CH_3_CN/0.1% trifluoroacetic acid and delivered to an Acclaim 300 (C18, 3 µm, 300 Å, 75 µm i.d. ×15 cm, Dionex Coorporation, CA) nanocolumn by a switching mechanism. Peptides were eluted from the nanocolumn at a flow rate of 250 nL/min with 2% CH_3_CN/0.1% formic acid (solvent A) and 90% CH_3_CN/0.1% formic acid (solvent B). The gradients used were: 0–30 min, 5% B (desalting); 30–80 min, 5–25% B; 80–95 min, 25–90% B; 95–110 min, 90% B; 110–120 min, 90–5% B; 120–130 min, 5% B.

A nanospray voltage in the range of 2000–2400 V was optimized daily. All nano LC MS/MS data were acquired in data-dependent acquisition mode in Analyst QS 1.1 (AB Sciex, Ontario, Canada). TOF MS survey scans with an m/z range of 300–1600 m/z for 1 s, followed by a product ion scan with an m/z range of 50–1600 m/z for 2 s each. Collision energy was automatically controlled by the IDA CE Parameters script.

### 2.7 Protein Identification

Once obtained, peak lists were generated from MS/MS spectra using AB SCIEX MS Data Converter ver 1.2 and searched against the IPI-Human database (version 3.73) concatenated with a reverse decoy using Mascot (version 2.3, Matrix Science). Fixed modification of cysteines to S-carbamidomethyl derivatives and variable oxidation (M) were defined for the database search. One missed cleavage was allowed with trypsin, and mass tolerance was set to 100 ppm for precursor ions and 0.2 Da for fragment ions. Searched results were exported as Mascot DAT files and grouped to protein matches using ProteoIQ (Ver.2.6.03, Nusep). Protein hits were filtered to include only those that were identified with at least 2 peptides with less than 1% false discovery rate. DAT files generated from the Mascot searches were exported to ProteoIQ ver. 2.3.06 for protein grouping and quantitation. For quantitation, the raw LC-MS/MS files converted to mzXML with Trans Proteomic Pipeline ver 4.4. Precursor intensities extracted from mzXML using area under the curve were matched to identify peptides for protein quantitation. Peak intensity ratios were calculated after the baseline was extracted after smoothing. The product of the square of the correlation coefficients (R^2^) was measured between the theoretical and experimental isotopic distributions for the quantitative precursors. Presence of an interfering precursor which overlaps with the distribution of interest were detected and removed from quantitation using the coefficient. Systematic bias was corrected with normalization factors. The normalization factors are calculated such that the total intensity for all peptides in each replicate is equal. The normalization factors were then applied to the ion intensities for each peptide. Identified proteins were relatively quantified using ^15^N metabolically labeled proteins as internal standards. P-values for the quantitation were generated by t-test using means of quantity, standard deviation and degrees of freedom based on the number of detected peptides. Identified proteins were annotated with Gene Ontology (GO) terms using Panther Classification system [14]. Protein list with relative quantitation and GO annotation is reported in Table S1.

### 2.8 Cell culture

Primary hepatocytes were isolated from mice at the Animal Models, Stem Cells, and Cell Therapy Core at the Marion Bessin Liver Research Center of Albert Einstein College of Medicine using protocol as described previously [15], [16]. After isolation cells were cultured in RPMI medium plus 10% FBS, 10^−7^ M dexamethasone, 10 µg/mL insulin and 5 µg/mL transferrin. 2.5×10^6^ cells were seeded onto a collagen I coated plate (60 (diameter)×15 (height) mm) (Becton Dickinson Labware) and incubated at 37°C for 4 hours. Then medium was replaced with fresh medium. Cells were treated with MGF (0 or 0.2 mM) for 24 hours, and then were collected in RIPA buffer for protein extraction.

### 2.9 Western blot

Protein samples were fractioned on 10% SDS-PAGE and transferred to polyvinylidene difluoride membrane. The membrane was incubated in 5% milk at room temperature for 1 hr, and subsequently incubated with anti-Dhtkd1 (Thermo Scientific Pierce, Cat# PA5-24208, 1∶150 dilution), anti-Cox6b1 (abcam, Cat # ab110266, 1∶1000), anti-Acac polyclonal antibody (Cell signaling, Cat.# 3676, 1∶1000) or anti-Scd1 (Cell signaling, Cat.# 2283, 1∶1000) in 5% milk (0.5–1 µg/ml) at 4°C overnight. The membrane was washed three times, 10 minutes each time, before incubated with secondary antibody, goat anti-rabbit IgG HRP-linked antibody in wash buffer with 0.5% milk (1∶2000). Secondary antibody was detected using SuperSignal West Pico Chemiluminescent Substrate (Thermo Scientific) followed by autoradiography. The protein bands were quantified using ImageJ.

### 2.10 Statistical analysis

Group measurements were expressed as average ± SD or average ± SEM. Comparisons between two groups were analyzed by Student's t tests. All statistical tests were two-sided and p<0.05 was considered significant.

## Results

### 3.1 MGF mitigates HFD induced lipid accumulation in plasma and liver and improves liver functions

**Figure 2:**
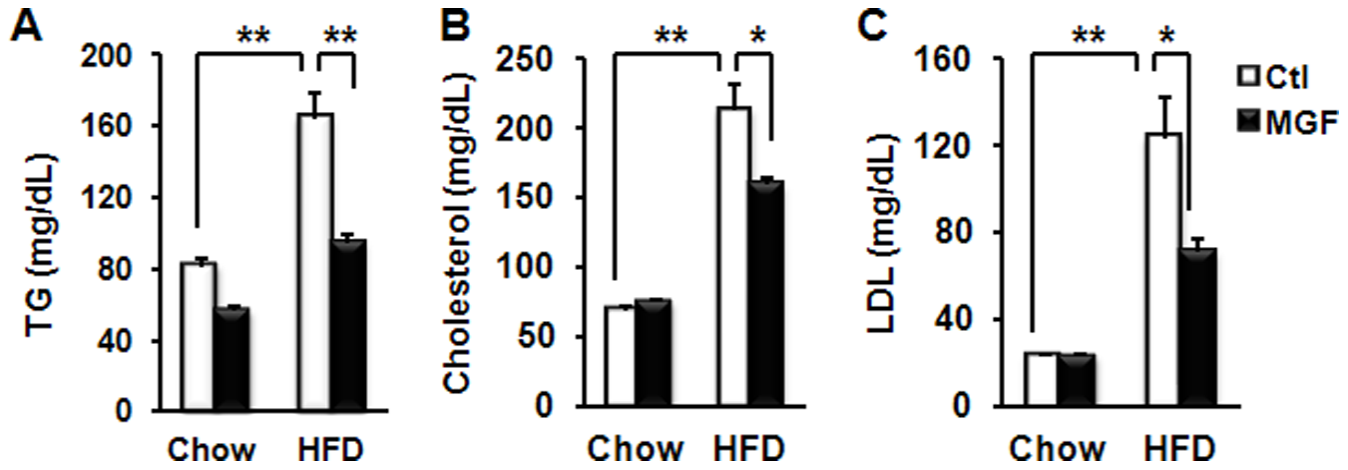
MGF mitigates HFD perturbed plasma lipids. Plasma triglyceride (TG) (A), cholesterol (B) and LDL (C) in C57BL6/J mice fed with chow or HFD ± MGF for 18 weeks. Values are average ± SEM (n = 8). *p<0.05, **p<0.01 by two tail t-test.

To investigate MGF modulation of HFD-induced detrimental effects in metabolism, we fed C57BL/6J mice, which are sensitive to HFD [17], with normal chow or HFD with MGF at dose of 0.00%, 0.25% or 0.50% (g/g food). Along with other phenotypic effects such as adiposity and insulin resistance, we observed that HFD feeding caused a 2–6 fold increase in TG, cholesterol and LDL in blood of mice, as compared to chow feeding (Figure 2). Treatment with 0.5% MGF resulted in 50% reduction in cholesterol and LDL in HFD-fed groups. Remarkably, MGF reversed HFD-induced increase in TG to the level similar to that measured in chow-fed mice.

**Figure 3:**
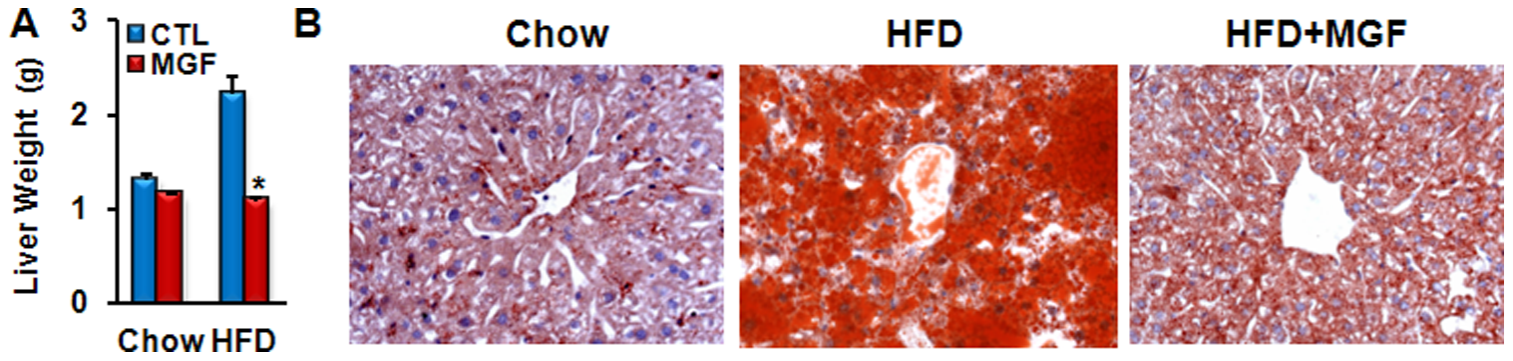
MGF prevents HFD induced liver enlargement and liver steatosis. Weight (A) and oil red O staining (B) of liver tissues of C57BL6/J mice fed with chow or HFD ± MGF for 18 weeks. Values are average ± SEM (n = 8). *p<0.05 by two tail t-test.

**Figure 4:**
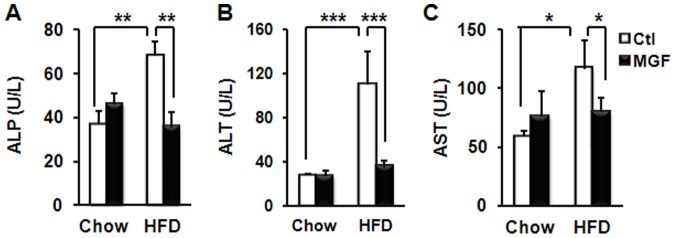
MGF improves liver functions and reduces HFD-induced liver toxicity. Plasma ALP (A), ALT (B) and AST (C) of C57BL6/J mice fed with chow or HFD ± MGF for 18 weeks. Values are average ± SEM (n = 8). *p<0.05, **p<0.01, ***p<0.001 by two tail t-test.

Since liver is the major organ where lipid metabolism occurs, we speculated that MGF targeted liver metabolism, and thus, examined liver tissues of mice fed with chow or HFD ± MGF. HFD feeding doubled liver weight as compared to chow feeding (Figure 3A). Impressively, MGF reduced liver weight of HFD-fed mice to the level lower than that of chow-fed mice (Figure 3A). Histological examination revealed that HFD feeding resulted in massive lipid accumulation (Figure 3B), which was almost completely eliminated by MGF treatment. TG is the major lipid stored in hepatocytes [18], and Oil Red O staining can faithfully visualize TG, as opposed to cholesterol, accumulation [19], [20]. The results shown in Figures 2 and 3 are in agreement with each other. Together they indicate that MGF has profound effects on lipid accumulation in the liver. In addition, it significantly improved liver functions impaired by HFD, as marked by plasma ALP, ALT and AST (Figure 4). Since these are also markers of liver toxicity, the results shown in Figure 4 indicate that MGF treatment did not cause any liver toxicity in the chow-fed group; rather it reduced HFD-induced toxicity.

### 3.2 Quantitative proteomic comparison of HFD + MGF vs. HFD using ^15^N labeled mouse livers

To determine the effects of MGF on global protein expression in liver tissues, we harvested liver tissues from 4 mice in each of two groups, HFD and HFD + MGF. Liver tissues from these mice were mixed with equal amounts of liver tissues from ^15^N labeled mice as illustrated in Figure 1. After separation of proteins by SDS-PAGE, 40 bands were in-gel digested prior to analysis by LC-MS/MS. Relative quantitation of protein expression levels in liver from mice treated with HFD + MGF was carried out using proteins in liver from ^15^N labeled mice as internal controls. Protein expression levels in liver from mice treated with HFD were also quantified with the identical internal controls. Ratios of proteins of HFD + MGF liver to those of HFD liver are depicted in Figure 1. This round of experiment was repeated once. From two rounds (4 mice/group/round) of reproducible experiments, we detected 965 proteins from all data sets and quantitatively compared 865 proteins between groups. The averaged ratios of each group are listed in Table S1.

### 3.3 MGF induces major changes in expression levels of proteins involved in metabolic processes

**Figure 5:**
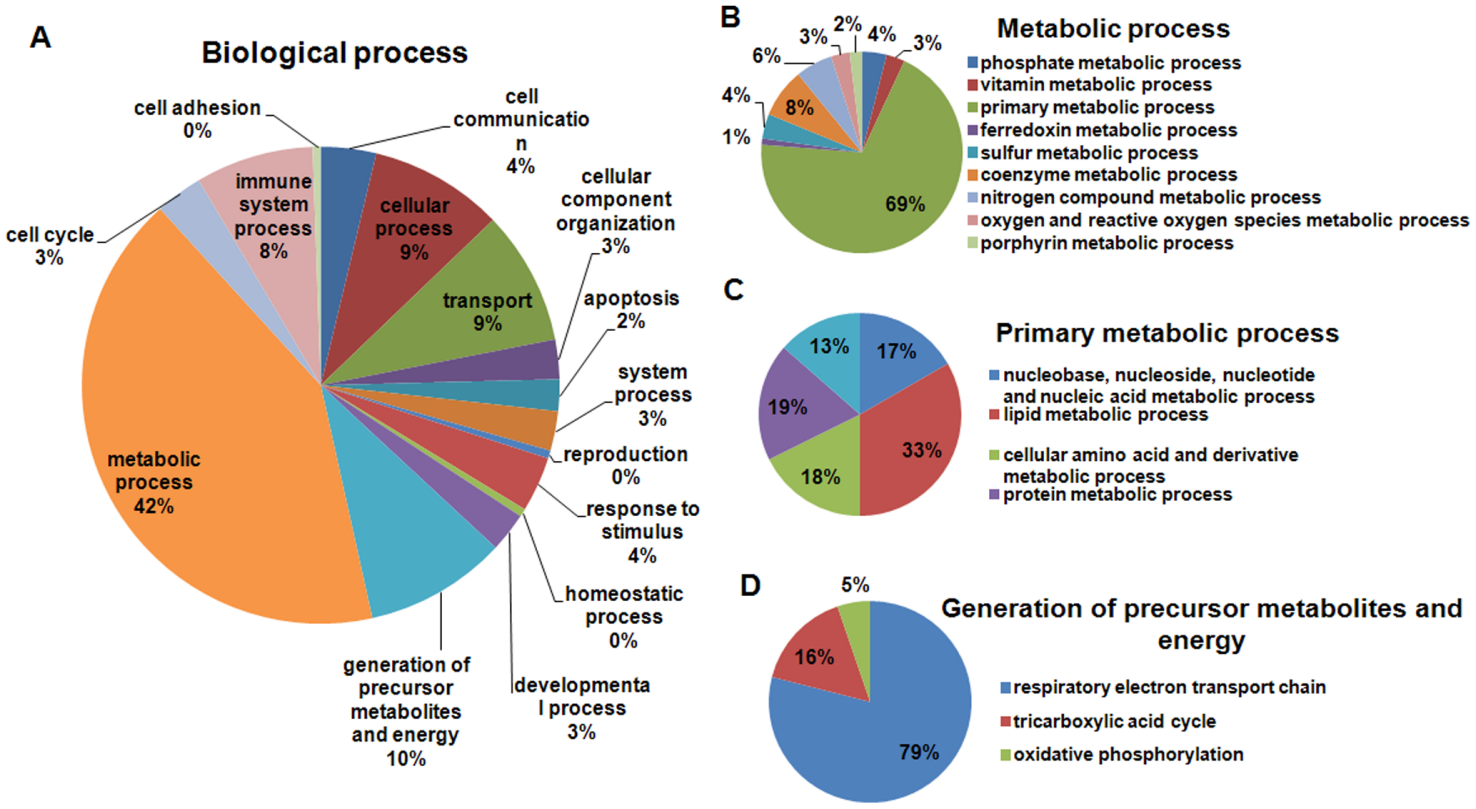
Classification of MGF modulated proteins according to biological processes using Gene Ontology (GO) analysis.

Among the 865 quantitatively compared proteins, 87 were downregulated or upregulated by MGF by more than 20% with p<0.05. They are presented in Table 1 in order from the most upregulated to the most downregulated. They were functionally annotated with GO terms. The distribution of these proteins among biological functions is illustrated in Figure 5A. It is noteworthy that about 50% of differentially regulated proteins are involved in two major actions, metabolic processes and generation of precursor metabolites and energy. The proteins involved in metabolic processes were further classified into 9 sub-classes of 9 metabolic processes (Figure 5B). Sixty nine percent of the proteins participate in primary metabolic processes (Figure 5B). The largest percentage of proteins in this category is involved in metabolic processes involving lipid transformations (Figure 5C). The proteins classified into the group of generation of precursor metabolites and energy metabolism were further divided into three groups involved in respiratory electron transport chain (ETC) (15 proteins), tricarboxylic acid (TCA) cycle (3 proteins) and oxidative phosphorylation (1 protein) (Figure 5D).

**Table 1:** MGF differentially regulates proteins in liver of mice fed with HFD.

Identifier	Gene symbol	Description	log_2_(HFD+MGF/^15^N)			log_2_(HFD/^15^N)			log_2_(HFD+MGF/HFD)	
			p∓	Avg.^a^	SD	p∓	Avg.^a^	SD	p^*^	Avg.^b^
IPI00269481	*Capzb*	Isoform 2 of F-actin-capping protein subunit beta	0.015	0.53	0	0.001	−1.98	0.06	0.000	2.51
IPI00756386	*Dhtkd1*	Probable 2-oxoglutarate dehydrogenase E1 component DHKTD1, mitochondrial	0	0.63	0.1	0.007	−1.04	0.12	NA	1.67
IPI00109257	*Cyp4f14*	Leukotriene-B4 omega-hydroxylase 3	0.022	0.73	0.16	0.023	−0.83	0.18	0.000	1.56
IPI00117991	*Cyp4f18*	Leukotriene-B(4) omega-hydroxylase 2	0.022	0.73	0.16	0	−0.81	0.29	0.000	1.54
IPI00221636	*2810007J24Rik*	sulfotransferase-like protein 1	0	2.46	0.69	0.02	1.05	0.93	0.007	1.41
IPI00128287	*Cyp1a2*	Cytochrome P450 1A2	0	−0.5	0.13	0	−1.8	0.26	0.000	1.3
IPI00128489	*Cyp2c50*	Isoform 1 of Cytochrome P450 2C50	0.004	0.77	0.06	0.029	−0.21	0.27	0.000	0.98
IPI00677085	*Prhoxnb*	2-oxo-4-hydroxy-4-carboxy-5-ureidoimidazoline decarboxylase	0	−0.87	0.02	0	−1.79	0.72	0.007	0.92
IPI00128271	*Kmo*	Isoform 1 of Kynurenine 3-monooxygenase	0	1.3	0.24	0	0.5	0.22	0.000	0.8
IPI00225390	*Cox6b1*	Cytochrome c oxidase subunit 6B1	0.001	0.85	0.56	0.013	0.06	0	0.001	0.79
IPI00620362	*Hnrnpl*	Heterogeneous nuclear ribonucleoprotein L	0.006	0.23	0.02	0.012	−0.53	0.21	0.000	0.76
IPI00120457	*Fdps*	Farnesyl pyrophosphate synthase	0	−1.13	0.55	0	−1.86	0.56	0.003	0.73
IPI00163011	*Txndc5*	Thioredoxin domain-containing protein 5	0.001	0.45	0.08	0.027	−0.28	0.24	0.000	0.73
IPI00131478	*Sult1a1*	Arylsulfotransferase ST1A4	0	−0.9	0.27	0	−1.6	0.31	0.000	0.7
IPI00228583	*Mtpn*	Myotrophin	0.003	−0.7	0.05	0.001	−1.4	0.05	0.000	0.7
IPI00471246	*Ivd*	Isovaleryl-CoA dehydrogenase, mitochondrial	0.002	0.33	0.32	0.028	−0.27	0.29	0.000	0.6
IPI00223714	*Hist1h1e*	Histone H1.4	0	0.75	0.3	0	0.17	0.09	0.000	0.58
IPI00758024	*Prdx6*	Putative uncharacterized protein	0.048	0.23	0.65	0	−0.35	0.23	0.000	0.58
IPI00165854	*Ube2n*	Ubiquitin-conjugating enzyme E2 N	0.033	−0.17	0.13	0	−0.73	0.1	0.000	0.56
IPI00454201	*Ccbl2*	Isoform 2 of Kynurenine-oxoglutarate transaminase 3	0	−0.91	0.28	0	−1.46	0.55	0.050	0.55
IPI00877205	*Got1*	aspartate aminotransferase, cytoplasmic	0	−0.54	0.64	0	−1.07	0.37	0.000	0.53
IPI00226993	*Txn1*	Thioredoxin	0	−0.33	0.1	0	−0.84	0.16	0.000	0.51
IPI00313236	*Slc27a5*	Bile acyl-CoA synthetase	0.028	0.32	0.55	0	−0.19	0.2	0.002	0.51
IPI00352984	*Xdh*	Xanthine dehydrogenase/oxidase	0.013	0.26	0.52	0.025	−0.25	0.54	0.008	0.51
IPI00555023	*Gstp1*	Glutathione S-transferase P 1	0	1.62	0.54	0	1.12	0.54	0.024	0.5
IPI00322156	*Slc38a3*	Sodium-coupled neutral amino acid transporter 3	0.001	−0.47	0.02	0.04	−0.97	0.28	NA	0.5
IPI00129056	*Glyat*	Isoform 1 of Glycine N-acyltransferase	0.001	0.32	0.26	0.01	−0.17	0.28	0.000	0.49
IPI00117705	*Ddost*	Dolichyl-diphosphooligosaccharide-protein glycosyltransferase 48 kDa subunit	0.032	0.63	0.16	0.008	0.17	0.21	0.001	0.46
IPI00308328	*Cyp2f2*	Cytochrome P450 2F2	0.002	0.84	0.41	0	0.38	0.2	0.016	0.46
IPI00223713	*Hist1h1c*	Histone H1.2	0	0.63	0.27	0.004	0.18	0.1	0.000	0.45
IPI00420329	*Snrnp200*	Small nuclear ribonucleoprotein 200	0.001	0.27	0.09	0.017	−0.17	0.13	0.000	0.44
IPI00111908	*Cps1*	Carbamoyl-phosphate synthase [ammonia], mitochondrial	0	0.11	0.38	0	−0.33	0.55	0.000	0.44
IPI00135651	*Slc25a13*	Calcium-binding mitochondrial carrier protein Aralar2	0	0.76	0.34	0	0.33	0.15	0.000	0.43
IPI00420136	*Snx3*	sorting nexin-3	0.005	−0.21	0.1	0	−0.63	0.08	0.000	0.42
IPI00120123	*Dmgdh*	Dimethylglycine dehydrogenase, mitochondrial	0.001	−0.09	0.21	0	−0.51	0.32	0.000	0.42
IPI00122549	*Vdac1*	Isoform Pl-VDAC1 of Voltage-dependent anion-selective channel protein 1	0	0.61	0.2	0.006	0.19	0.25	0.000	0.42
IPI00467841	*Calm*	Putative uncharacterized protein	0.013	0.19	0.22	0.004	−0.22	0.18	0.001	0.41
IPI00322828	*Farsb*	Phenylalanyl-tRNA synthetase beta chain	0.028	0.27	0.14	0.003	−0.13	0	0.000	0.4
IPI00111412	*Rpl4*	60S ribosomal protein L4	0	0.59	0.37	0.002	0.19	0.2	0.001	0.4
IPI00172146	*Dhdh*	Trans-1,2-dihydrobenzene-1,2-diol dehydrogenase	0.011	−0.21	0.17	0	−0.6	0.3	0.000	0.39
IPI00129577	*Aifm1*	Apoptosis-inducing factor 1, mitochondrial	0	0.68	0.23	0	0.3	0.18	0.000	0.38
IPI00408961	*Haao*	3-hydroxyanthranilate 3,4-dioxygenase	0.001	−0.18	0.18	0	−0.55	0.43	0.001	0.37
IPI00230351	*Sdha*	Succinate dehydrogenase [ubiquinone] flavoprotein subunit, mitochondrial	0	0.51	0.4	0.002	0.14	0.28	0.000	0.37
IPI00223216	*Tst*	Thiosulfate sulfurtransferase	0.009	0.17	0.36	0.014	−0.19	0.39	0.004	0.36
IPI00132728	*Cyc1*	Isoform 1 of Cytochrome c1, heme protein, mitochondrial	0	0.68	0.25	0.002	0.33	0.26	0.005	0.35
IPI00112322	*Ugt2b5*	Putative uncharacterized protein	0.006	0.68	0.22	0	0.33	0.25	0.042	0.35
IPI00136213	*Sardh*	Sarcosine dehydrogenase, mitochondrial	0	0.15	0.48	0	−0.2	0.61	0.002	0.35
IPI00109275	*Slc25a22*	Mitochondrial glutamate carrier 1	0	0.59	0.23	0.045	0.24	0.26	0.018	0.35
IPI00115482	*Slc10a1*	Sodium/bile acid cotransporter	0.001	−0.14	0.05	0	−0.49	0.16	NA	0.35
IPI00331251	*Acads*	Acyl-Coenzyme A dehydrogenase, short chain, isoform CRA_a	0	0.51	0.35	0.029	0.17	0.31	0.000	0.34
IPI00230507	*Atp5h*	ATP synthase subunit d, mitochondrial	0	0.66	0.4	0	0.33	0.18	0.003	0.33
IPI00308162	*Slc25a12*	Calcium-binding mitochondrial carrier protein Aralar1	0	0.67	0.32	0	0.34	0.15	0.007	0.33
IPI00274175	*Gm5121*	40S ribosomal protein S8	0	0.45	0.28	0.025	0.12	0.13	0.007	0.33
IPI00123223	*Mug1*	Murinoglobulin-1	0	0.48	0.22	0.002	0.15	0.21	0.000	0.33
IPI00341282	*Atp5f1*	ATP synthase subunit b, mitochondrial	0	0.57	0.34	0.04	0.24	0.33	0.009	0.33
IPI00129928	*Fh1*	Isoform Mitochondrial of Fumarate hydratase, mitochondrial	0	0.38	0.18	0.031	0.06	0.08	0.000	0.32
IPI00126625	*Acsm1*	Isoform 1 of Acyl-coenzyme A synthetase ACSM1, mitochondrial	0.009	0.16	0.28	0.008	−0.16	0.43	0.013	0.32
IPI00153317	*Aldh1l1*	10-formyltetrahydrofolate dehydrogenase	0	−0.21	0.56	0	−0.52	0.47	0.000	0.31
IPI00131177	*Letm1*	LETM1 and EF-hand domain-containing protein 1, mitochondrial	0	0.53	0.27	0.011	0.22	0.22	0.006	0.31
IPI00121985	*Slco1b2*	Isoform 1 of Solute carrier organic anion transporter family member 1B2	0	0.62	0.17	0	0.31	0.08	0.007	0.31
IPI00169463	*Tubb2c*	Tubulin beta-2C chain	0.004	−0.19	0.3	0	−0.5	0.45	0.022	0.31
IPI00122862	*Mthfd1*	C-1-tetrahydrofolate synthase, cytoplasmic	0	−0.27	0.21	0	−0.58	0.42	0.000	0.31
IPI00330363	*Rpl7a*	60S ribosomal protein L7a	0	0.58	0.34	0.002	0.28	0.25	0.007	0.3
IPI00138691	*Arpc4*	Actin-related protein 2/3 complex subunit 4	0.012	−0.17	0.07	0.001	−0.46	0.31	0.012	0.29
IPI00223217	*Rpl13a*	60S ribosomal protein L13a	0	0.45	0.08	0	0.17	0.02	0.000	0.28
IPI00313222	*Rpl6*	60S ribosomal protein L6	0	0.38	0.39	0.009	0.1	0.12	0.008	0.28
IPI00131424	*Cpt2*	Carnitine O-palmitoyltransferase 2, mitochondrial	0	0.68	0.27	0	0.41	0.29	0.010	0.27
IPI00112549	*Acsl1*	Long-chain-fatty-acid-CoA ligase 1	0	1.06	0.49	0	0.79	0.39	0.003	0.27
IPI00622780	*Aco1*	Cytoplasmic aconitase	0	−0.18	0.31	0	−0.45	0.47	0.005	0.27
IPI00136505	*Gm4953*	Uncharacterized protein	0.015	0.53	0	0.03	0.26	0	NA	0.27
IPI00454049	*Echs1*	Enoyl-CoA hydratase, mitochondrial	0.005	0.33	0.42	0.047	0.1	0.1	0.034	0.23
IPI00469380	*Aox3*	Aldehyde oxidase 1	0	1.14	0.35	0	0.91	0.55	0.000	0.23
IPI00112139	*Ociad2*	OCIA domain-containing protein 2	0.004	1.39	0.39	0.009	1.68	0.23	NA	−0.29
IPI00462072	*Eno1*	Alpha-enolase	0.001	−0.58	0.29	0	−0.28	0.39	0.038	−0.3
IPI00114840	*Endog*	Endonuclease G, mitochondrial	0	−0.72	0.01	0.013	−0.42	0.24	0.013	−0.3
IPI00129512	*Psmb4*	Proteasome subunit beta type-4	0.048	−0.54	0.17	0.03	−0.17	0.04	0.000	−0.37
IPI00331628	*Hsd17b4*	Peroxisomal multifunctional enzyme type 2	0	0.4	0.25	0	0.78	0.33	0.000	−0.38
IPI00127206	*Aldob*	Fructose-bisphosphate aldolase B	0	−0.1	0.54	0	0.28	0.98	0.021	−0.38
IPI00135189	*Aacs*	Acetoacetyl-CoA synthetase	0.007	−2.06	0.24	0	−1.57	0.39	0.049	−0.49
IPI00126248	*Acly*	ATP-citrate synthase isoform 1	0	−2.17	0.18	0	−1.64	0.37	0.000	−0.53
IPI00130144	*Ephx1*	Microsomal epoxide hydrolase	0.045	0.23	0.38	0	0.77	0.39	0.000	−0.54
IPI00121833	*Acaa1b;Acaa1a*	3-ketoacyl-CoA thiolase A, peroxisomal	0	0.53	0.59	0	1.2	0.51	0.000	−0.67
IPI00122139	*Acaa1b;Acaa1a*	3-ketoacyl-CoA thiolase B, peroxisomal	0	0.56	0.64	0	1.28	0.47	0.000	−0.72
IPI00474783	*Acaca*	Isoform 1 of Acetyl-CoA carboxylase 1	0	−2.17	0.43	0	−1.34	0.29	0.000	−0.83
IPI00421241	*Acacb*	acetyl-Coenzyme A carboxylase beta	0	−2.22	0.47	0	−1.37	0.45	0.000	−0.85
IPI00128692	*Nsdhl*	Sterol-4-alpha-carboxylate 3-dehydrogenase, decarboxylating	0.012	−0.41	0.06	0.031	0.55	0.3	0.000	−0.96
IPI00322530	*Scd1*	Acyl-CoA desaturase 1	0.049	−2.43	0.79	0.032	−1.11	0.29	NA	−1.32

a average of several peptides from two rounds; ^b^average of two ratios from two rounds.

∓ probability of precision for each ratios; ^*^probability of accuracy at means based on the degree of freedom using number of detected peptides.

SD, standard deviation; NA, not sufficient degree of freedom due to limited peptide numbers.

Among proteins involved in generation of precursor metabolites and energy, MGF increased oxoglutarate dehydrogenase E1 (Dhtkd1) (+3.2 fold), cytochrome c oxidase subunit 6B1 (Cox6b1) (+1.7 fold), fumarate hydratase 1 (Fh1) (+1.2 fold), short-chain specific acyl-CoA dehydrogenase (Acads) (+1.3 fold), enoyl-CoA hydratase (+1.2 fold) and carnitine O-palmitoyltransferase 2 (Cpt2) (+1.2 fold). MGF also increased calcium-binding mitochondrial carrier protein (+1.3 fold), bile acyl-CoA synthetase (+1.4 fold), glutathione S-transferase P1 (Gstp1) (+1.4 fold). In addition, MGF increased levels of several isoforms of cytochrome P450, cytochrome P450 2F2 (+1.4 fold), cytochrome P450 2C50 (+2.0 fold), and cytochrome P450 1A2 (+2.5 fold). Among proteins participating in lipid metabolic processes, MGF reduced fatty acid stearoyl-CoA desaturase 1 (Scd1) (−2.5 fold) and acetyl-CoA carboxylase 1 (Acac1) (−1.8 fold).

### 3.4 Orthogonal validation of proteomic data

**Figure 6:**
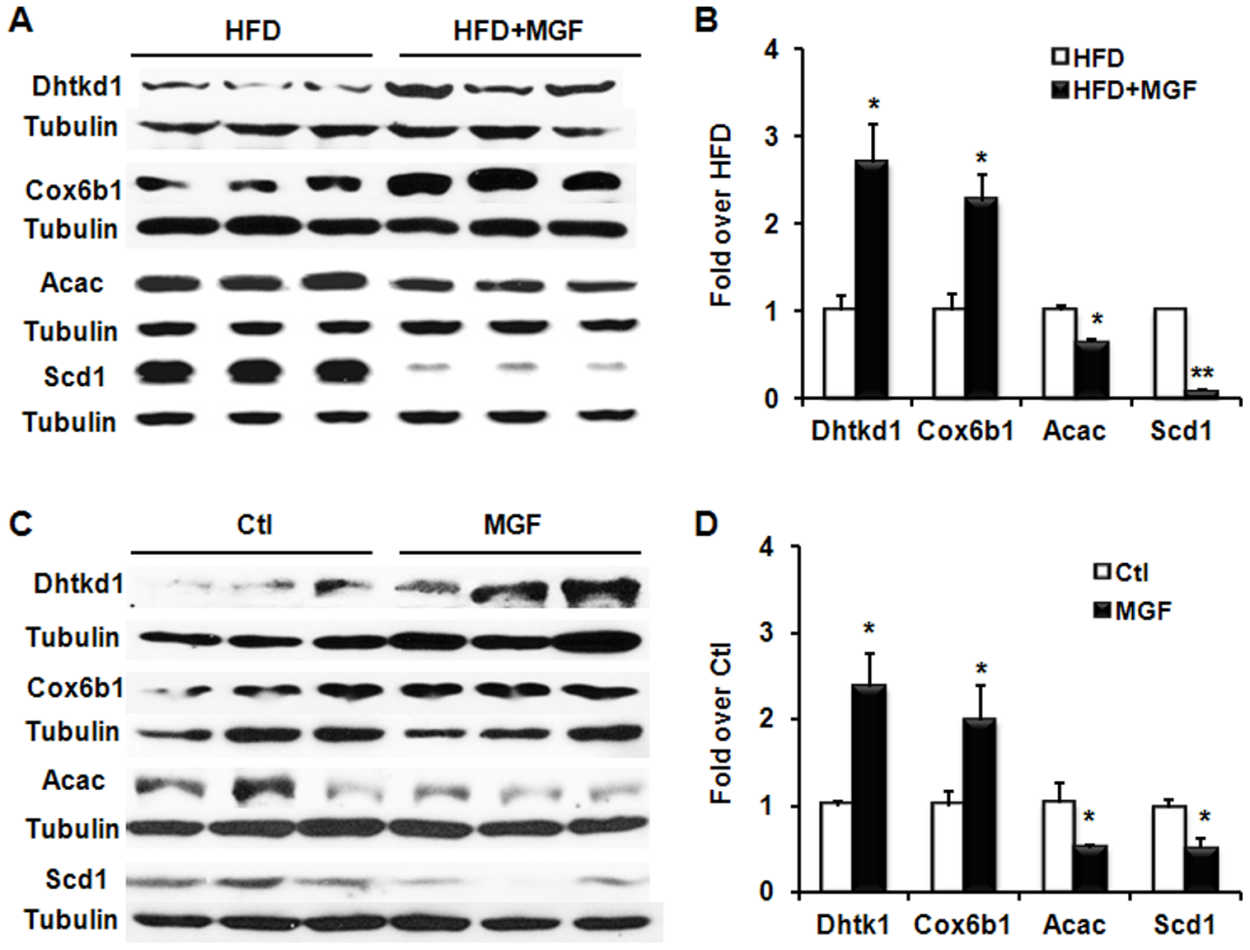
MGF induces Dhtkd1 and Cox6b, and suppresses Acac and Scd1 in hepatic tissues and cells. (A) Western blots of Dhtkd1, Cox6b1, Acac and Scd1 in proteins extracted from liver tissues from C57BL6/J mice fed with HFD ± MGF for 18 weeks. (B) Quantification of protein expression in (A). Liver tissues were from 2 groups (HFD ± MGF), 3 mice per group. Values are average ± SEM (n = 3). (C) Western blots of Dhtkd1, Cox6b1, Acac and Scd1 in proteins extracted from mouse primary hepatocytes and treated with or without 200 µM MGF for 24 hours. (D) Quantification of protein expression in (C). Values are average ± SEM (n = 3). *p<0.05, **p<0.01 by two tail t-test.

MGF exerts a high impact on mitochondrial proteins, such as Dhtkd1 and Cox6b1, and proteins in lipid metabolism including Scd1 and Acac. Therefore, we chose these four proteins to further confirm and validate the proteomics data. We assayed the expression levels of Dhtkd1, Cox6b1, Scd1 and Acac in liver tissues from mice treated with HFD ± MGF by western blot. As shown in Figure 6A and 6B, MGF significantly increased Dhtkd1 and Cox6b1 by 270% and 230%, respectively. Conversely, it reduced Acac to 65% of controls, and reduced Scd1 to less than 9% of controls. These changes confirm and validate results of proteomic data. To determine whether these MGF effects occur in a cell autonomous manner, we treated primary hepatocytes isolated from mice with or without 200 µM MGF for 24 hours and determined protein expression levels by western blot. Patterns of protein changes (Figure 6C, 6D) are similar to those in liver tissues, suggesting that MGF directly modulates the abundance of these four proteins independent of organism tissue or hormonal effects. In summary, MGF has direct influence on proteins participating in mitochondrial bioenergetics and lipid synthesis at the hepatocyte level.

### 3.5 MGF is hypothesized to enhance mitochondrial bioenergetics and suppress lipogesis

**Figure 7:**
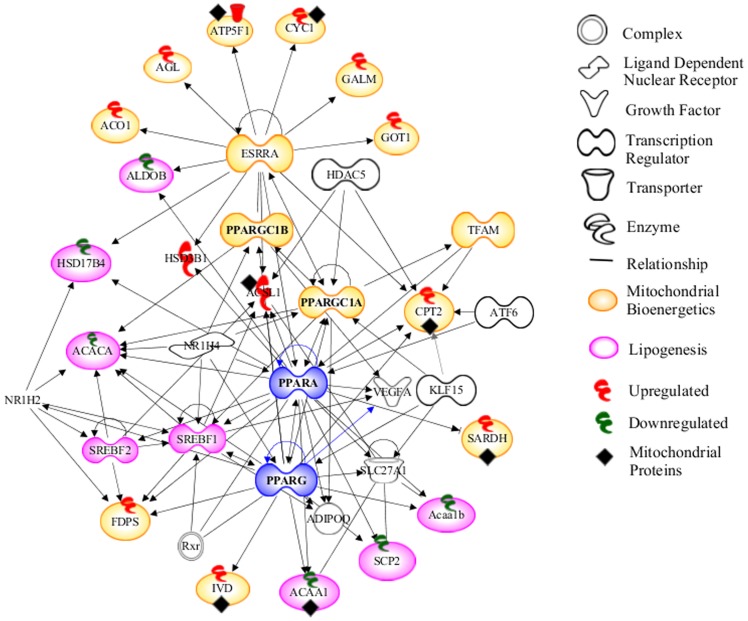
Network of MGF modulated proteins and predicted MGF modulated pathways by Ingenuity Pathway Analysis.

All significantly differentially modulated proteins by MGF were submitted to the ingenuity pathway analysis (IPA). The resulting network shown in Figure 7 illustrates that MGF upregulates proteins in oxidative mitochondrial bioenergetics pathway, which are networked to important transcriptional factors transcription factor A, mitochondrial (Tfam), and estrogen-related receptor alpha (Esrrα), and their cofactors peroxisome proliferator-activated receptor-γ coactivator (PGC)-1α and PGC-1β [21]–[25]. Concurrently MGF downregulates several proteins in the lipogenesis pathway, which are networked to the transcriptional factor, sterol regulatory element binding protein-1 (SREBP-1). All of these factors are connected to peroxisome proliferator-activated receptor alpha (PPARα) and PPARγ, which regulates a wide array of genes involved in lipid metabolism and energy homeostasis [26]. The pathway analysis of this system study predicts that MGF modulates metabolism via a dual mode of pharmacodynamics action, upregulation of mitochondrial bioenergetics and downregulation of lipogenesis.

## Discussion

The use of metabolically labeled mammalian tissues as internal references has been proposed to be a powerful approach to quantitatively analyze the proteome-wide changes that can occur in an organism in response to a drug [27]. Here, we report the first quantitative proteomics study of the global impact of a natural compound, MGF, on proteome of liver of mice fed with HFD, utilizing mouse liver tissue labeled with ^15^N, i.e. SILAM liver. In the SILAM method, proteins of the entire mice are labeled via incorporation of ^15^N by feeding mice with ^15^N-labeled fortified diet [27]. When mixed with the liver of HFD- or HFD + MGF-fed mice, this SILAM liver generates hundreds of thousands of isotopically labeled peptides in comparable amounts for mass spectrometry based proteomic analysis. As shown in Table S1, we have successfully generated a database of 865 liver proteins, of which 87 are significantly altered by MGF, demonstrating the impact of MGF on liver protein expression at a global scale.

This unbiased systemic study reveals novel findings that MGF significantly upregulates mitochondrial biogenesis by increasing several important proteins in the TCA cycle and the ETC, including Dhtkd1, Cox6b1, Fh, Acads and Cpt2 (Table 1, Figure 5D, 7), some of which was further confirmed by protein immuno-blots (Figure 6).

Dhtkd1 (E1) is the first subunit of a multienzyme system, 2-oxoglutarate dehydrogenase complex (Ogdh). Dhtkd1 is essential for mitochondrial biogenesis and function maintenance [60], as it initiates the substrate-specific and irreversible stage of the overall reaction catalyzed by Ogdh [28], [29]. Ogdh is a key regulatory site within the TCA cycle, controlling the supply of reducing equivalents (NADH) to the ETC, leading to ATP production [30], [31]. In addition, this enzyme utilizes amino acids for energy production, and produces succinyl-CoA for heme biosynthesis [28]. Deficiency in Dhtkd1 causes DOOR syndrome [32]. Impairment of Ogdh resulted from mutation causes neurological symptoms and early death in humans [33], [34] [61], [62]. Animals with null mutants of the OgdhC are non-viable [29], [35], [36]. Here we show that MGF can increase Dhtkd1 protein level in both liver tissues and cultured primary hepatocytes (Table 1, Figure 6), which could lead to elevation of NADH flux into the ETC. Interestingly, MGF increases the abundance of the complexes in the ETC, particularly, complex IV, cytochrome c oxidase (Cox), presumably to accommodate the elevated NADH flux.

Cox is composed of 13 subunits, one of which is Cox6b1. Cox6b1 is incorporated in the very last step of Cox assembly. Mutation in Cox6b1 causes mitochondrial encephalomyopathy due to Cox deficiency [37]. Cox is the terminal complex of the mitochondrial respiratory chain, the ultimate acceptor of all the reducing equivalents derived from the breakdown of sugars, amino acids, and fatty acids [38], [39]. Cox deficiency is the most frequent cause of respiratory chain defects in humans, which could lead to the most severe mitochondrial diseases [38], [39], including Leigh syndrome, hepatic failure and encephalomyopathy [38]. MGF is able to increase this enzyme (Table 1, Figure 6), presumably-improving liver mitochondrial activity.

Fh is an enzyme that catalyzes the reversible hydration/dehydration of fumarate to malate. It has two isoforms: the mitochondrial isoenzyme involved in the TCA cycle, and the cytosolic isoenzyme involved in the metabolism of amino acids and fumarate [40]. The observations of hereditary leiomyomatosis and renal cell carcinoma (HLRCC) syndrome in humans with Fh mutations have linked mitochondrial metabolism to cancer [41]–[44]. Deficiency in mitochondrial Fh and or other mitochondrial enzymes including succinate dehydrogenase(Sdh) disables mitochondrial oxidative phosphorylation and consequently enhances anaerobic utilization of carbohydrates, which provides the energy source supporting cancer cell viability under hypoxic conditions. Accumulation of fumarate in the cytosol due to dysfunction of cytosolic Fh increases the stability of hypoxic inducing factor, which stimulates tumorigenesis [45]. MGF upregulates both Fh and Sdh, suggesting that MGF should be investigated as a potential cancer suppressor.

In addition to these mitochondrial proteins, MGF upregulates Gstp1 expression by 1.4 fold, indicating that MGF might provide antioxidative effects and protect liver against oxidative stress. These effects could be partially contributory to MGF protection against peroxidation [6], [46], [47].

In contrast, MGF downregulates a number of proteins involved in lipid metabolism (Figure 5C). For example we confirmed by both proteomics analyses and immunoblot that MGF significantly reduced Acac and Scd1 (Table 1, Figure 6), two enzymes critical for *de novo* lipogenesis.

Acac catalyzes the irreversible carboxylation of acetyl-CoA to produce malonyl-CoA, the rate-determining step in fatty acid synthesis [48]. Liver-specific deletion of Acac1 reduces hepatic triglyceride accumulation without affecting glucose homeostasis [49]. Previous studies have shown that MGF suppresses Acac1 in liver of hamster and rat, and in human hepatic cells (HepG2) [9], [10]. Our unbiased proteomics and specific immunochemistry data show that MGF substantially reduces Acac1 in mouse hepatic tissues and cells (Table 1) (Figure 6), indicating that the effect of MGF on Acc1 crosses several species. Further, the present study extends our understanding of the impact of MGF on lipogenesis, as we show that MGF drastically suppressed another important protein in lipogenesis, Scd1 (Figure 6).

Scd1 catalyzes the conversion of stearoyl-CoA to oleoyl-CoA, which is a major substrate for TG synthesis. Mice with global deletion of Scd1 are resistant to HFD and genetically induced obesity [50], [51]. Global Scd1 deletion also prevents the liver steatosis observed in a number of mouse models, including high-carbohydrate diet- and HFD-fed mice, ob/ob, PPARα-/- and aP2-SREBP-1c lipodystrophic mice [50]–[53]. Scd1 is the protein in lipid metabolism most profoundly downregulated by MGF (Table 1). The effect of MGF on Scd1 examined by western blot appears to be even greater (Figures 6A and 6B). It has been shown that Scd1 deficiency reduces mRNA of SREBP-1 [54], a master regulator of lipogenesis [55]. Most of genes in the lipogenesis pathway are targets of SREBP-1 [56]-[58]. It is conceivable that MGF could be a suppressor of SREBP-1, which is predicted by IPA of our MS data (Figure 7). This prediction is supported by the report by Guo et al. showing that MGF treatment in hamster resulted in reduced mRNA of SREBP-1c in liver [9].

In addition to SREBP1, Guo et al. also showed that MGF increased mRNA of PPARα [9], again, validating the prediction by the network shown in Figure 7. This network also predicts that MGF upregulates transcription of genes in mitochondrial biogenesis. Albeit further investigation is needed to confirm these predictions, this network allows us to generate hypothesis that MGF upregulates oxidative mitochondrial bioenergetics pathways and downregulates lipogenesis pathways. Uniquely, these dual effects of MGF enable it to increase energy expenditure and suppress lipid production and ultimately prevent diet induced hyperlipidemia, hyperglycemia and insulin resistance.

MGF is a small molecular with molecular weight less than 500. Structurally it possesses fewer than 5 donor functions for hydrogen bonds and fewer than 10 acceptor functions for hydrogen bonds, which satisfies Lipinski's rules for druglike properties and fulfills the requisites for high bioavailability by oral administration. Although the present study was conducted in mice and the expression profiles between mouse and human may differ, our additional data obtained in cultured human hepatocytes (data not shown), as well as the data shown by others [10], strongly suggest that MGF could exert similar effects on those proteins in humans. The potential application of MGF as a nutraceutical or pharmaceutical agent benefiting human health is supported by a recent report that mango extracts containing MGF as the main component induces weight loss in overweight and obese humans, accompanied by improved fasting blood glucose and lipid profiles [59].

## Conclusion

This unbiased proteomics study reports quantitative measures of proteome-wide changes caused by MGF. It provides novel and comprehensive information on the effects of MGF on protein expression in mouse liver, and provides insight into the molecular mechanisms by which MGF protects against HFD induced liver steatosis and other obesity related metabolic disorders. Specifically, MGF upregulates proteins participating in mitochondrial bioenergetics and downregulates proteins controlling *de novo* lipogenesis. This novel mode of dual pharmacophore actions may enable MGF to enhance energy expenditure and inhibit lipogenesis, and thereby eliminate liver steatosis and prevent adiposity. It also strongly suggests that MGF has potential to be developed into a therapeutic agent preventing diet induced metabolic disorders.

## Supporting Information

Table S1 **Quantitative analysis of relative protein expression levels in liver of mice treated with high fat diet in the presence and absence of mangiferin by stable isotope labeling of mammalians (SILAM) method.**
(XLSX)
