# Correction: Dual Mode Action of Mangiferin in Mouse Liver under High Fat Diet

**DOI:** 10.1371/journal.pone.0100170

**Published:** 2014-06-17

**Authors:** The PLOS ONE Staff

The first two authors, Jihyeon Lim and Zhongbo Liu, should be noted as contributing equally to this work, not as corresponding authors. The publisher apologizes for the error.

The sole corresponding author is Yuling Chi, who can be contacted at yuling.chi@einstein.yu.edu.
